# The Business of T Cell Subsets and Cytokines in the Immunopathogenesis of Inflammatory Bowel Disease

**DOI:** 10.7759/cureus.27290

**Published:** 2022-07-26

**Authors:** Shreekant Bharti, Mridushri Bharti

**Affiliations:** 1 Pathology/Laboratory Medicine, All India Institute of Medical Sciences Patna, Patna, IND; 2 Microbiology, Netaji Subhas Medical College and Hospital, Patna, IND

**Keywords:** crohn’s, ulcerative, colitis, t cell, cytokines, inflammatory bowel disease

## Abstract

Inflammatory bowel disease (IBD) is a chronic inflammatory disorder and one of the most common inflammatory diseases of gastrointestinal (GI) tract in young adults. It is now equally prevalent in western countries as well as in Asian countries. Recently, there has been an increasing IBD burden in low- to middle-income countries as opposed to the earlier notion of this being a disease of the affluents. It occurs due to a variety of factors, namely, local immune alteration, disruption and inflammation of the mucosa, environmental factors, microbial commensals, and pathogen-induced genetic predisposition or genetic alteration in protective factors, etc.

So far, an exact etiopathogenesis of IBD is yet to be completely elucidated. Several recent types of research have emphasized the role of altered innate and humoral immunity in its causation, many of them based on animal models of IBD. Due to the poor understanding of its etiopathogenesis, IBD is still a challenge for the treating clinicians leading to persistent and recurrent disease in many cases.

Immune dysregulation in the GI tract incited by various pathogenic stimuli has gained great attention from researchers in the field of IBD. This review focuses on highlighting the role of various T cell subsets, their interplay, and associated cytokines involved in the pathogenesis of IBD along with a short description of genetic as well as other immunological factors. A better understanding of the pathogenic factors and subsequent randomized controlled trials targeting these factors is prudent for better therapeutic approaches for IBD.

## Introduction and background

Introduction

Inflammatory bowel disease (IBD) generically represents a chronic inflammatory disorder involving the gastrointestinal (GI) tract that majorly includes two forms of diseases, namely, Crohn’s disease (CD) and ulcerative colitis (UC) [1]. The key characteristic of these two conditions is an onset of the disease typically during young adulthood with a prolonged period of relapse and remission [2]. The etiology of IBD is not clear as there is a complex interplay of several factors such as immunological, genetic, environmental, microbiological, and a complicated regulatory mechanism in association with the intestinal mucosal immunity that impacts the development and onset of the disease [3].

UC presents as mucosal inflammation, particularly involving the colon, while CD manifests as patchy inflammation within the proximal colon, mainly the ileum [4]. Both CD and UC are complex diseases where a wide range of genetic loci facilitates disease susceptibility. Besides genetic factors, the role of microbiological and environmental factors has also been identified owing to a high incidence of IBD observed in identical twins (50% and 10% in CD and UC, respectively) [5]. An altered microflora within the GI tract is a key contributing factor to increasing IBD susceptibility [4].

IBD is under the influence of the expression of various cytokines, their receptors, growth factors, and proteins involved in molecular pathways such as interleukin (IL)-23R, IL-12B, positive regulatory domain I element (PRDM1), Janus kinase 2 (JAK2), signal transducer and activator of transcription 3 (STAT3), signal transducer and activator of transcription 4 (STAT4), and Ikaros family zinc finger protein 1 (IKZF1). All these pathways indicate the critical role played by IL-12/IL-23 and T helper (Th)1/Th17 cells [6]. In addition, other susceptible genes are also involved in the progression of UC and CD including suppressor of mothers against decapentaplegic (SMAD)3, SMAD7, and SMAD ubiquitination-related factor (SMURF), the genes regulating signaling of transforming growth factor (TGF) [7]. Recent evidence suggests a defect in immunosuppressive cytokines in association with UC and CD such as IL-10 [8].

Studies have shown that Th cells play a significant role in the pathogenesis of the disease, where Th1 cells influence the severity of intestinal inflammation, particularly in the case of CD. On the other hand, Th2 cells are believed to be actively participating in UC [9]. Recent evidence demonstrates engagement of Th17 cells and impairment of regulatory T cells (Tregs) as an important component regulating the intestinal immune balance [10]. Therefore, various immune subsets of CD4+ T cells and other immune cells have a crucial role in the development of IBD including Th1, Th2, Th17, and Tregs. The disease progression is largely attributed to an immunological imbalance between the Th cells and Tregs, which disrupts the immune homeostasis [9]. Tregs' immune regulation properties promote the expression of forkhead box P3 (FOXP3) transcription factor and CD25, all of which are responsible for immune tolerance due to their immunosuppressive abilities [11]. Recent research also highlights the role of certain novel pathogenic lymphoid cells also known as innate lymphoid cells (ILCs) contributing to the pathogenesis of IBD.

The present review aims to elaborate on the role of CD4+ Th cells and other immune cells involved in the pathogenesis of IBD. This will help to assess the Th lineage-specific transcription factors (TFs) and associated cytokines involved in the etiopathogenesis of IBD [12].

Background

IBD is a chronic inflammatory disorder and one of the most common inflammatory diseases of the GI tract in young adults. It is now equally prevalent in western countries as well as in Asian countries. Recently, there has been an increasing IBD burden in low- to middle-income countries as opposed to the earlier notion of this being a disease of the affluents. It occurs due to a variety of factors, namely, local immune alteration, disruption and inflammation of the mucosa, environmental factors, microbial commensals, and pathogen-induced genetic predisposition or genetic alteration in protective factors, etc.

So far, an exact etiopathogenesis of IBD is yet to be completely elucidated. Several recent pieces of research have emphasized the role of altered innate and humoral immunity in its causation, including a few animal models of IBD. Immune dysregulation in the GI tract incited by various pathogenic stimuli has also gained great attention from researchers in the field of IBD. Various T cell subsets like Th1, Th2, Th17, Tregs, and associated cytokines such as IL-1, IL-2, IL-12 IL-23, etc. with their corresponding receptors have been found to be responsible for mucosal immune dysregulation and subsequent disease development. Due to the poor understanding of its etiopathogenesis, IBD is still a challenge for the treating clinicians leading to persistent and recurrent disease in many cases.

This review focuses on highlighting the role of various T cell subsets, their interplay, and associated cytokines involved in the pathogenesis of IBD along with a short description of genetic as well as other immunological factors. A better understanding of the pathogenic factors and subsequent randomized controlled trials targeting these factors is prudent for better therapeutic approaches for IBD.

## Review

Pathogenesis of IBD

**Figure 1:**
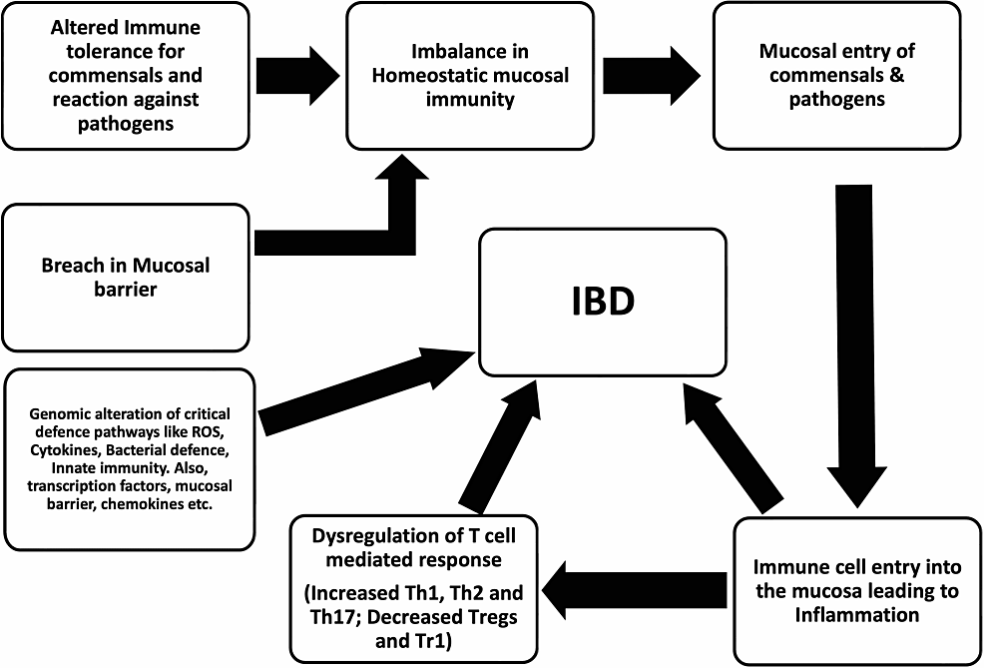
Simplified schema of immunological factors involved in the causation of IBD IBD: Inflammatory bowel disease; ROS: Reactive oxygen species; Th: T helper; Tr1: Regulatory T cell type 1.

In IBD, inflammation occurs within the intestine, which acts as the main site for disease development [13]. Intestine consists of a diverse and dense amount of commensal microbiota, making it a unique environment for bacteria and other pathogens. The immune system within the intestine is engaged in a complex process of homeostatic immunity [14]. Balanced homeostasis provides a symbiotic relationship between the host and commensal. This is required for the maintenance of a healthy gut and, eventually, for the health of the host [15]. Homeostatic immunity regulates the interaction between the host and bacteria by two crucial mechanisms. First is the level of stability and immune tolerance toward commensals and potential pathogens. Second is the integrity of the intestinal mucosal barrier in regulating the protective environment within the gut [16]. Loss of mucosal barrier compromises the structural framework that is ensured by the mucus layer produced by the goblet cells and a single layer of epithelial cells. This facilitates the entrance of luminal microbial products in addition to the commensal bacteria that penetrate the subjacent lamina propria and therefore results in the activation of an inflammatory immune response [17]. As a result, innate and adaptive cells infiltrate the intestinal mucosa and accentuate the disruption of the mucosal barrier in IBD. In this process of inflammation, effector T cells and dysregulation of T cell-mediated tolerance are identified as major components contributing to the onset and progress of this condition [14]. A simplified diagram to explain the different factors involved in the pathogenesis of IBD has been depicted in Figure 1.

Expression of Th1 and Th17 has been identified in both UC and CD patients, although variation in Th2 responses distinguishes CD from UC [8]. Patients with IBD have an increased percentage of mucosal cytokines produced by Th17 and Th1/Th17 cells [18]. Studies have shown that patients with inflamed mucosa have an increased activity of IL-22 and IL-17A/IL-23 axis in both CD and UC. A similar pattern of the disease has been observed in experimental animal models with artificially enhanced IL-22 and IL-17A/IL-23 levels. Furthermore, reduced responses of FOXP3 Tregs and regulatory T cell type 1 (Tr1) and increased responses of Th1, Th2, and Th17 are believed to play a crucial role in the pathogenesis of IBD [8,19].

As mentioned earlier, balanced mucosal immunity is integral to maintaining immune homeostasis and defense [20]. The main immune cells required for intestinal immunity are effector CD4+ or CD8+ T cells, dendritic cells (DCs), macrophages, and Tregs. Active participation of different cell types is the main reason accounting for heterogeneous effects and disease manifestations in relation to IBD. Genome-wide association evidence reports more than 100 specific loci responsible for IBD development with critical pathways, including reactive oxygen, innate immune response, microbial defense, antimicrobial activity, and intestinal barrier [21].

To gain a better understanding of the underlying mechanism of IBD, several animal models have been studied [22]. Mice models have been popularly used to assess the role of bacterial, genetic, and other specific factors contributing to the onset of IBD [23]. Studies on mouse models have been conducted in all kinds of environmental settings such as germ-free and xenobiotic mice to identify the importance of specific microflora [19]. Additionally, experimental mouse models or genetic models have been developed to gain insights into the etiology of IBD.

Role of cytokines

Various IBD-associated genes have been associated to be strongly involved in disease progression. The main factor is the role of CD4+ Th cells in the onset and development of IBD, which affects the normal intestinal barrier function [24]. The pathways interact in a suitable manner to minimize the risk of IBD; however, impaired intestinal homeostasis augments it. Cytokines play an important part in regulating these pathways, thus promoting molecular interaction between the epithelium, microbiota, and mucosal immune cells. This shows that cytokines and numerous cytokine receptors are among the most strongly associated class of genes regulating IBD such as IL-1R1, IL-2, IL-2RA, IL-12B, IL-27, IL-12R, and IL-23R [24].

Currently, the pathogenic role of Th1 and Th17 cells in UC and CD is suggested to be associated with the occurrence of the p40 subunit as a common factor related to certain key cytokines including IL-23, IL-12, and heterodimeric cytokines. IL-23 promotes the expression of Th17 cells and inhibits the Tregs response, eventually facilitating gut inflammation with increased activity of the IL-23/IL-17 axis in IBD [25]. In recent times, growing evidence demonstrates the importance of cytokines in innate immunity and maintenance of epithelial functions. T cells and, especially, CD4+ Th cells play a crucial role in this disease. A short recapitulation of the cytokines, chemokines, and TFs involved in the pathogenesis of IBD has been listed in Table 1.

**Table 1:** A concise list of cytokines, chemokine receptors, adhesion molecules, and transcription factors involved in the pathogenesis of IBD IBD: Inflammatory bowel disease; Th: T helper; IL: Interleukin; TNF: Tumor necrosis factor; TGF: Transforming growth factor; CCR: Chemokine receptor; CD: Cluster of differentiation; T-bet: T-box expressed in T cells; STAT: Signal transducer and activator of transcription; GATA: GATA transcription factor; NF-kB: Nuclear factor kappa-light-chain-enhancer of activated B cells; NOD2: Nucleotide-binding oligomerization domain-containing protein 2.

Cytokine activity in IBD	Chemokine receptors and adhesion molecules in IBD	Transcription factors in IBD
Increased mucosal cytokines secreted by Th17, Th1/Th17 cells	CCR4, CCR5, CCR7, CCR9, CD62L, aEb7 integrin, a4b7 integrin	T-bet, STATs, GATA3, NF-kB, NOD2
Increased activity of IL-22 and IL-17A/IL-23 axis		
Other cytokines: IL-1b, IL-2, IL-2Ra, IL-10, IL-12B, IL-12R, IL-15, IL-16, IL-18, IL-22, IL-23R, IL-27, TNF-alpha, TGF-beta		

An understanding of Th cells and other immune cells provides an insight into the genetics of cytokine dysregulation and relevant treatment options that can be used for its management by developing therapeutics designed for tissue-specific functions [4].

CD4+ T cells and T cell subsets in IBD

Bone marrow precursor cells produce Th cells, while in the thymus, a specific T cell antigen receptor (TCR) is acquired by these cells such that they enter peripheral circulation including the blood and lymphatic vessels. The activation of naïve Th cells occurs in the peripheral lymphoid organs such as the spleen and peripheral lymph nodes where they are recognized by unique major histocompatibility complex class II (MHC-II) expressed by DCs. Furthermore, the activation of Th cells stimulates clonal expansion along with differentiation of the cells into distinct phenotypes that represent a wide number of effector and regulatory subsets [26]. The variants usually depend on the cytokines present in the microenvironment. The Th subsets include a list of functional cell types [26], which are (i) Th1 cells that express interferon-gamma (IFNγ); (ii) Th2 cells that secrete IL-4; (iii) Th22 cells that express IL-22; (iv) Th17 cells that secrete IL-17A, IL-17F, IL-22, and “induced” Tregs (iTregs); (v) Th9 cells that secrete IL-9; and (vi) T follicular helper (Tfh) cells that secrete IL-21 and control germinal center activities in lymphoid organs.The functions of all these Th cells are maintained by the polarized expression of regulatory or effector cytokines.

The exact role of Th cells in UC and CD remains controversial. Particularly, the specificity of T cell response is under the influence of the MHC; however, the significance of genetic susceptibility is more significant in UC than in CD. For example, in certain chemically induced mouse models, T cell involvement was not identified in IBD [24]. On the other hand, few chronic mouse models were Th cell-dependent such as SAMP1/YitFc ileitis [4] and T cell transfer-induced colitis.

Role of T Helper 1 (Th1) Cells

Th1 cells are an integral component that provides effective protection against infectious pathogens. These are mainly associated with the production of tumor necrosis factor (TNF) and IFNγ, which in turn directly activate cytotoxic CD8+ T cells and macrophages, respectively. The combined action of these cytokines promotes the destruction of pathogenic bacteria and viruses [27]. Patients with IBD have higher Th1 cells accumulated in their intestinal tract, particularly in CD individuals that directly influence the disease. The antigen-presenting cells produce IL-12 that plays a major role in promoting the differentiation of naïve T cells into Th1 cells through the signal transducer and activator of transcription 4 (STAT4) [28].

Studies conducted in mice have shown that Th1/Th17 bias in IBD may be caused due to the specific microenvironment in the ileum so that even in the absence of inflammation, there is a promotion of Th1 and Th17 cell development. Various mouse models have demonstrated the pathogenic potential of Th1 cells. Moreover, Th cells obtained from STAT4^−/^ mice were unable to transfer intestinal inflammation in recipients with severe combined immunodeficiency [29].

Role of Th2 Cells 

Th2 cells are considered important effectors when it comes to airway inflammation in asthma [30]. Studies have mentioned high levels of Th2-related cytokines, especially IL-5 and IL-13 in intestinal immune cells of lamina propria of patients with UC. In addition, reports also demonstrate increased levels of GATA binding protein 3 (GATA-3), a Th2 master transcription factor in IBD patients [31].

Th2 cells produce IL-5, a major cytokine that regulates differentiation of eosinophil (in bone marrow) and tissue infiltration. The role of IL-5 and eosinophil in IBD is unclear, although a recent study conducted in a mouse model reported the influence of IL-5 and granulocyte-macrophage colony-stimulating factor (GM-CSF) in increasing the eosinophil activation and tissue damage, which were predominantly dependent on IL-23. These findings are of paramount importance as they demonstrate the influence of various Th cell subtypes (Th17/GM-CSF, Th2/IL-5) and how they can enhance mucosal inflammation [4]. However, the involvement of various subsets of Th2 cells such as IL-446 and other associated cytokines remains controversial. Further studies are required to establish its clear role in the pathogenesis of IBD.

Th17 Cells in IBD 

The traditional approach includes a Th1/Th2 paradigm in both UC and CD, which was later challenged with the introduction of IL-17-expressing Th17 cells [23]. Th17 cells play a significant part in restoring the commensal population and promoting epithelial barriers at sites such as the skin and gut. However, conditions, where the immune system is impaired disease, can exacerbate [32].

The differentiation of Th17 is largely regulated by IL-6 and TGF-β and enhanced by accessory cytokines such as IL-23 and IL-1β [33]. Th17 differentiation is downregulated by cytokines including IFN-γ, IL-4, and IL-2 [34]. There are two different forms of Th17, mainly the pathogenic and non-pathogenic forms. The pathogenic Th17 cells exhibit a special feature as they produce both IFN-γ and IL-17A [35]. The production of IL-17A/IFNγ by T cells is one of the important characteristics of IBD patients, demonstrating the development of inflamed intestinal tissue. This unique feature is also seen in other conditions such as rheumatoid arthritis (RA) and psoriatic skin lesions [36]. On the contrary, non-pathogenic Th17 cells express IL-17A with IL-10 and lack the expression of IFN-γ. Pathogenic Th17 cells have a more specific transcriptional profile than non-pathogenic Th17 cells and are expressed by higher levels of IL-23 receptor (IL-23R). The strongest genetic association with CD is shown by IL-23R, together with NOD2, denoting the fact that IL-23R regulates the signaling in IBD patients [37]. Interestingly, Th17-signature genes are seen to be closely associated with human IBD with common genes presented by them such as the granulopoiesis cytokine GM-CSF (colony-stimulating factor 2 [CSF2]), Th1-associated TFs (T-bet [Tbx21],STAT4), the IL-7 receptor alpha chain (Il7r), and granzyme B (Gzmb) [24].

Regulatory T Cells (Tregs)

Regulatory T cells consist of heterogeneous subtypes of CD4+ T cells, exhibiting immunosuppressive properties. These cells demonstrate an increased level of FOXP3, a master transcription factor, and IL-2 receptor (CD25), which are among the most significant populations related to immunosuppressive phenotype [38]. FOXP3 presents with various immunosuppressive activities against intestinal inflammation, infections, tumors, autoimmune diseases, and allergies. It has two main subsets, FOXP3-negative type 1 Treg (Tr1) cells and FOXP3-positive Tregs.

Tregs play a crucial role in establishing gut immune homeostasis and regulating the inflammatory response exhibited by innate and adaptive immune effectors [39]. Mouse models with severe autoimmune phenotype in addition to the genetic defect of the FOXP3 gene prevent the development of Tregs resulting in impaired activation of the gut immune system [40] and eventually leading to inflammation in the gut. Furthermore, evidence shows that a mutation in the FOXP3 gene contributes to the development of immune dysregulation, polyendocrinopathy, enteropathy, X-linked (IPEX) syndrome, a rare autoimmune disease, along with other autoimmune conditions such as IBD, diabetes, and arthritis [41]. IBD patients generally have a higher amount of Tregs in the inflamed mucosal epithelium compared to the non-inflamed mucosal part. The functions and phenotype of the regulatory helper cells differ in the inflamed mucosa of IBD patients from that of peripheral lymphoid organs of individuals in a healthy state. Patients with IBD have decreased number of peripheral Tregs and a higher amount of peripheral Th17 cells [42]. Furthermore, patients with IBD demonstrate higher expression of FOXP3 and increased levels of cytokines such as IL-17A, IL-1, and IL-6 [34]. Additionally, Tregs are associated with the tissue repair mechanism within the intestine. Considering all kinds of populations of intestinal Treg, it is observed that Tr1 cells are actively involved in the repair of the intestinal mucosa, especially where co-expression of master TFs, GATA3 and Th2, occurs [43]. Table 2 highlights the major alterations in Th and regulatory cells' activity in different IBDs and the common genomic pathways that contribute to their pathogenesis.

**Table 2:** Summary of predominant T cell type activity and the common genomic pathway alterations contributing to the pathogenesis of IBD IBD: Inflammatory bowel disease; Th: T helper; Tr1: Regulatory T cell type 1; UC: Ulcerative colitis; CD: Crohn’s disease.

T cell activity in IBD	Genomic alterations in IBD (approx. > 100 loci) for critical protective pathways
Increased response of Th1, Th2, Th17	Reactive oxygen species
Decreased response of Treg and Tr1	Cytokine receptors
UC: Th2 predominant	Innate immune responses
CD: Th1 predominant	Microbial defense
	Antimicrobial activity
	Mucosal barrier of intestine

The findings based on various clinical studies implicate the role of Tregs in controlling mucosal inflammation, although altered Treg function remains an issue of concern. Few studies have reported a decreased number of these cells in the inflamed mucosa of patients with IBD [41], while others have mentioned a higher expression of FOXP3 [44]. The underlying mechanism associated with Treg-mediated immune suppression needs further evaluation as several molecules have been linked with the regulation of intestinal immune balance around Treg cells [45].

ILCs in IBD

ILCs ensure the protective immunity within the mucosal tissues. They are a recently identified class of effector lymphocytes that produce important cytokines involved in IBD. ILCs are unique due to the absence of antigen-specific receptors and phenotypic characteristics in association with immune cells, although they have specific lymphoid morphology [46]. The cells can be categorized into three distinct subsets depending on the expression of TFs associated with lineage differentiation, which include ILC1, ILC2, and ILC3.

The key ILCs implicated in the pathogenesis of IBD are the ones that express IL-17 and IFN-γ. A *Helicobacter hepaticus *IBD model demonstrated the pathogenicity of ILC3 [47]. Additionally, IBD patients also express innate immune cells linked to ILC3 genes (for example, IL-17A, IL-22, and IL-23R) [48]. Patients with CD have higher levels of IFN-γ-producing ILC1. Another important factor seen in CD patients is an increased amount of IL-12 and IL-15 expressed in relation to intraepithelial CD103+NKp46+ILC1 and NKp46+ILC1 expressed in lamina propria, highlighting a significant role in the pathogenesis of the IBD within the ileum [49].

To gain a better understanding of the underlying mechanism of IBD, several animal models have been studied [21]. Mice models have been popularly used to assess the role of bacterial, genetic, and other specific factors contributing to the onset of IBD [22]. Studies on mouse models have been conducted in all kinds of environmental settings such as germ-free and xenobiotic mice to identify the importance of specific microflora [23]. Additionally, experimental mouse models or genetic models have been developed to gain insights into the etiology of IBD (Table 3) [19].

**Table 3:** Short review of the animal models of IBD pathogenicity Rag-/-/SCID: Recombination activating genes knockout model/severe combined immunodeficiency; TNF: Tumor necrosis factor; UC: Ulcerative colitis; TLR: Toll-like receptors; Treg cells: Regulatory T cells; CD40LTG: CD40 ligand transgene; IFNγ: Interferon-gamma; IBD: Inflammatory bowel disease.

Animal model	Agent implied	Outcome
Chemical drug-induced model	DSS (Dextran sulfate sodium)	TNF-α, IL-17, and reduced Treg cells suggestive of increased inflammation in the intestine epithelium.
	TNBS (2,4,6-trinitrobenzene sulfonic acid)	IL-12, IL-17, and reduced Treg cells suggestive of increased inflammation in the intestine epithelium.
	Indomethacin	Reduced prostaglandin E1 and E2. This resulted in increased levels of reactive oxygen species (ROS) and a few other free radicals, eventually causing IBD.
	Oxazolon	Increased production of IL-4 and IL-5 driven by Th2. This shows an association similar to human UC.
Adoptive transfer model	Naïve T cell→Rag^-/-^/SCID	Recovery by Treg cell transfer as seen in IBD patients.
	CD8 transfer	DNBS led to colitis, with the production of cytotoxic CD8+ T cell (Tc1) by IFN. The disease was inhibited by depleting the antibody of CD8+ and no other variants of CD4+ T cells.
Bacteria-infected model	*C. rodentium* acute infection	IL-17 as an active component of IBD.
	Helicobactor hepaticus	The mice models infected with *H. hepaticus* exhibited CD45RB high T cells, suggesting abnormal immune response in IBD.
Gene-manipulated model	IL-2^-/-^	Reduced Treg cells indicating increased intestinal inflammation.
	IL-10	Reduced Treg cells indicating increased intestinal inflammation.
	Myd88	Impaired TLR signal indicating increased intestinal inflammation.
	Mdr1a^-/-^	Reduced iTreg cell differentiation shows impaired immune balance.
	CD4/PDK1^-/-^	Reduced Treg cell indicating increased intestinal inflammation.
	TNFΔARE	Increased TG and CD8 function suggestive of abnormal immune regulation.
	B/CD40LTG	Increased IFNγ indicating increased intestinal inflammation.
	TNBS	Low-dose IL-2 used as a therapeutic intervention for expanding Tregs.
	TNBS	Higher colonic inflammation and small bowel enteropathy due to transfer of CD4+ T cells in animal models.
	TNBS	Low-dose (LD) IL-2 used as a therapeutic intervention for expanding Tregs.

## Conclusions

In addition to other factors, immune cells and cytokines play a significant role in the pathogenesis of IBD. Understanding of the pathogenetic factors, especially immune regulation in IBD pathogenesis, has increased vastly in recent years. The important T cell subsets and other immune cells necessary for maintaining mucosal immunity, epithelial barrier, and overall gut health have been discussed in this review article. Findings obtained from several animal models emphasize that IBD therapy requires continued integration of genomic analysis, functional assessment, and identification of specific risk factors that lead to the onset of this chronic inflammatory disease. Immune dysregulation and its clear understanding will help to address this prevalent disease more precisely. Further randomized clinical trials should be conducted to gain insights into the underlying mechanism and develop advanced therapeutic strategies that can effectively treat IBD.

## References

[1] Fiocchi C (2005). Inflammatory bowel disease pathogenesis: therapeutic implications. Chin J Dig Dis.

[2] Baumgart DC, Sandborn WJ (2012). Seminar Crohn’s disease. Lancet.

[3] Huttenhower C, Kostic AD, Xavier RJ (2014). Inflammatory bowel disease as a model for translating the microbiome. Immunity.

[4] Chen ML, Sundrud MS (2016). Cytokine networks and T-cell subsets in inflammatory bowel diseases. Inflamm Bowel Dis.

[5] Loddo I, Romano C (2015). Inflammatory bowel disease: genetics, epigenetics, and pathogenesis. Front Immunol.

[6] Huang H, Fang M, Jostins L (2017). Fine-mapping inflammatory bowel disease loci to single-variant resolution. Nature.

[7] Fowler SA, Ananthakrishnan AN, Gardet A (2014). SMAD3 gene variant is a risk factor for recurrent surgery in patients with Crohn's disease. J Crohns Colitis.

[8] Fu SH, Chien MW, Hsu CY, Liu YW, Sytwu HK (2020). Interplay between cytokine circuitry and transcriptional regulation shaping helper T cell pathogenicity and plasticity in inflammatory bowel disease. Int J Mol Sci.

[9] Tindemans I, Joosse ME, Samsom JN (2020). Dissecting the heterogeneity in T-cell mediated inflammation in IBD. Cells.

[10] Cheon JH (2013). Genetics of inflammatory bowel diseases: a comparison between western and eastern perspectives. J Gastroenterol Hepatol.

[11] Pereira LM, Gomes ST, Ishak R, Vallinoto AC (2017). Regulatory T cell and forkhead box protein 3 as modulators of immune homeostasis. Front Immunol.

[12] Lee SH, Kwon JE, Cho ML (2018). Immunological pathogenesis of inflammatory bowel disease. Intest Res.

[13] Däbritz J, Gerner P, Enninger A, Claßen M, Radke M (2017). Inflammatory bowel disease in childhood and adolescence. Dtsch Arztebl Int.

[14] Belkaid Y, Harrison OJ (2017). Homeostatic immunity and the microbiota. Immunity.

[15] Liu Y, Lan Q, Lu L (2014). Phenotypic and functional characteristic of a newly identified CD8+ FOXP3-CD103+ regulatory T cells. J Mol Cell Biol.

[16] Sprouse ML, Bates NA, Felix KM, Wu HJ (2019). Impact of gut microbiota on gut-distal autoimmunity: a focus on T cells. Immunology.

[17] Turpin W, Lee SH, Raygoza Garay JA (2020). Increased intestinal permeability is associated with later development of Crohn’s disease. Gastroenterology.

[18] Rovedatti L, Kudo T, Biancheri P (2009). Differential regulation of interleukin 17 and interferon gamma production in inflammatory bowel disease. Gut.

[19] Yamada A, Arakaki R, Saito M, Tsunematsu T, Kudo Y, Ishimaru N (2016). Role of regulatory T cell in the pathogenesis of inflammatory bowel disease. World J Gastroenterol.

[20] Krishnan K, Arnone B, Buchman A (2011). Intestinal growth factors: potential use in the treatment of inflammatory bowel disease and their role in mucosal healing. Inflamm Bowel Dis.

[21] Khor B, Gardet A, Xavier RJ (2011). Genetics and pathogenesis of inflammatory bowel disease. Nature.

[22] Corridoni D, Arseneau KO, Cominelli F (2014). Inflammatory bowel disease. Immunol Lett.

[23] Nell S, Suerbaum S, Josenhans C (2010). The impact of the microbiota on the pathogenesis of IBD: lessons from mouse infection models. Nat Rev Microbiol.

[24] Jostins L, Ripke S, Weersma RK (2012). Host-microbe interactions have shaped the genetic architecture of inflammatory bowel disease. Nature.

[25] Croxford AL, Kulig P, Becher B (2014). IL-12-and IL-23 in health and disease.

[26] Nakayamada S, Takahashi H, Kanno Y, O'Shea JJ (2012). Helper T cell diversity and plasticity. Curr Opin Immunol.

[27] Targan SR, Hanauer SB, van Deventer SJ (1997). A short-term study of chimeric monoclonal antibody cA2 to tumor necrosis factor alpha for Crohn's disease. Crohn's disease cA2 study group. N Engl J Med.

[28] Thierfelder WE, van Deursen JM, Yamamoto K (1996). Requirement for Stat4 in interleukin-12-mediated responses of natural killer and T cells. Nature.

[29] Claesson MH, Bregenholt S, Bonhagen K (1999). Colitis-inducing potency of CD4+ T cells in immunodeficient, adoptive hosts depends on their state of activation, IL-12 responsiveness, and CD45RB surface phenotype. J Immunol.

[30] Fahy JV (2015). Type 2 inflammation in asthma--present in most, absent in many. Nat Rev Immunol.

[31] Heller F, Florian P, Bojarski C (2005). Interleukin-13 is the key effector Th2 cytokine in ulcerative colitis that affects epithelial tight junctions, apoptosis, and cell restitution. Gastroenterology.

[32] Weaver CT, Elson CO, Fouser LA, Kolls JK (2013). The Th17 pathway and inflammatory diseases of the intestines, lungs, and skin. Annu Rev Pathol.

[33] Chung Y, Chang SH, Martinez GJ (2009). Critical regulation of early Th17 cell differentiation by interleukin-1 signaling. Immunity.

[34] Laurence A, Tato CM, Davidson TS (2007). Interleukin-2 signaling via STAT5 constrains T helper 17 cell generation. Immunity.

[35] Ghoreschi K, Laurence A, Yang XP (2010). Generation of pathogenic T(H)17 cells in the absence of TGF-β signalling. Nature.

[36] Cosmi L, Maggi L, Santarlasci V, Liotta F, Annunziato F (2014). T helper cells plasticity in inflammation. Cytometry A.

[37] Hue S, Ahern P, Buonocore S (2006). Interleukin-23 drives innate and T cell-mediated intestinal inflammation. J Exp Med.

[38] Li Z, Li D, Tsun A, Li B (2015). FOXP3+ regulatory T cells and their functional regulation. Cell Mol Immunol.

[39] Wildin RS, Ramsdell F, Peake J (2001). X-linked neonatal diabetes mellitus, enteropathy and endocrinopathy syndrome is the human equivalent of mouse scurfy. Nat Genet.

[40] Boehm F, Martin M, Kesselring R, Schiechl G, Geissler EK, Schlitt HJ, Fichtner-Feigl S (2012). Deletion of Foxp3+ regulatory T cells in genetically targeted mice supports development of intestinal inflammation. BMC Gastroenterol.

[41] Monticelli LA, Osborne LC, Noti M, Tran SV, Zaiss DM, Artis D (2015). IL-33 promotes an innate immune pathway of intestinal tissue protection dependent on amphiregulin-EGFR interactions. Proc Natl Acad Sci U S A.

[42] Veltkamp C, Anstaett M, Wahl K (2011). Apoptosis of regulatory T lymphocytes is increased in chronic inflammatory bowel disease and reversed by anti-TNFα treatment. Gut.

[43] Eastaff-Leung N, Mabarrack N, Barbour A, Cummins A, Barry S (2010). Foxp3+ regulatory T cells, Th17 effector cells, and cytokine environment in inflammatory bowel disease. J Clin Immunol.

[44] Josefowicz SZ, Lu LF, Rudensky AY (2012). Regulatory T cells: mechanisms of differentiation and function. Annu Rev Immunol.

[45] Spits H, Cupedo T (2012). Innate lymphoid cells: emerging insights in development, lineage relationships, and function. Annu Rev Immunol.

[46] Buonocore S, Ahern PP, Uhlig HH, Ivanov II, Littman DR, Maloy KJ, Powrie F (2010). Innate lymphoid cells drive interleukin-23-dependent innate intestinal pathology. Nature.

[47] Geremia A, Arancibia-Cárcamo CV, Fleming MP (2011). IL-23-responsive innate lymphoid cells are increased in inflammatory bowel disease. J Exp Med.

[48] Fuchs A, Vermi W, Lee JS (2013). Intraepithelial type 1 innate lymphoid cells are a unique subset of IL-12- and IL-15-responsive IFN-γ-producing cells. Immunity.

[49] Negi S, Saini S, Tandel N, Sahu K, Mishra RP, Tyagi RK (2021). Translating treg therapy for inflammatory bowel disease in humanized mice. Cells.

